# ‘The bones tell a story the child is too young or too frightened to tell’: The Battered Child Syndrome in Post-war Britain and America

**DOI:** 10.1093/shm/hkv040

**Published:** 2015-04-15

**Authors:** Jennifer Crane

**Keywords:** child abuse, post-war, radiology, medical authority, secrecy

## Abstract

This article traces the emergence of child abuse as a medical concern in post-war Britain and America. In the early 1960s American paediatricians and radiologists defined the ‘battered child syndrome’ to characterise infants subjected to serious physical abuse. In the British context, paediatricians and radiologists, but also dermatologists and ophthalmologists, drew upon this work and sought to identify clear diagnostic signs of child maltreatment. For a time, the x-ray seemed to provide a reliable and objective visualisation of child maltreatment. By 1970, however, medical professionals began to invite social workers and policy makers to aid them in the diagnosis and management of child abuse. Discourse around the ‘battered child syndrome’, specifically, faded away, whilst concerns around child abuse grew. The battered child syndrome was a brief phenomenon of the 1960s, examination of which can inform the histories of medical authority, radiology and secrecy and privacy in the post-war period.

In 1962 the paediatrician C. Henry Kempe published an article entitled ‘The Battered Child Syndrome’ in the *Journal of the American Medical Association*, which was co-authored by experts in paediatrics, psychiatry, obstetrics and gynaecology and radiology.^1^ The article characterised the battered child syndrome as ‘a clinical condition in young children who have received serious physical abuse, generally from a parent or foster parent’.^2^ The article sprung from Kempe's work at the University of Colorado Hospital, where he found that it was common practice to offer ‘patently absurd’ explanations for the injuries of children.^3^ Children who had clearly been beaten by their parents were regularly misdiagnosed with rare brittle bone diseases, ‘spontaneous’ cases of subdural haematoma, unexplained bleeding disorders, and with the catch-all notion of ‘failure to thrive’.^4^ The authors recognised that physicians were reluctant to believe that parents could inflict violence on their children, and that children often struggled to tell people that they were being mistreated. Kempe and his co-authors argued that the x-ray could provide radiologists and paediatricians with a clear confirmation of whether a child had been beaten. Their article stated: ‘To the informed physician, the bones tell a story the child is too young or too frightened to tell’.^5^

In this article I will highlight the role of medicine and particularly radiology in facilitating the emergence of social and political concern around child abuse in post-war Britain and America. First, I will explain that in the 1940s and 1950s American paediatricians and radiologists paid increasingly close attention to the discrepancies apparent between parental accounts and children's injuries. The idea that doctors should treat children as their patients, and challenge parents' explanations, was radical. Kempe's article drew upon these early pieces of research, but also invented the term ‘battered child syndrome’ and expounded the usage of x-rays. Nevertheless, there were limitations to the potential of the x-ray in diagnosing child maltreatment. The x-ray image could not provide clear information about how children's fractures had been created—whether accidentally or as the result of parental violence. X-ray images had to be ‘read’; interpreted by radiologists, paediatricians and general practitioners.

Second, the article will consider how the syndrome was received in Britain. Kempe's work quickly influenced a number of physicians from a range of fields, including radiologists and paediatricians and also dermatologists and ophthalmologists. These actors sought to further characterise the battered child syndrome. X-rays, again, were presented as a highly useful tool. Work on the battered child syndrome was published in medical journals and newspapers, and brought child maltreatment to the attention of policy makers and charities. The National Society for the Prevention of Cruelty to Children (NSPCC), directly inspired by Kempe, established a Battered Child Research Unit in 1967. Medicine quickly lost its monopoly in identifying and managing child maltreatment, as physicians invited social work professionals, teachers, families and neighbours to play a role in the protection of children. Child abuse became a social and political problem, as well as a medical one. The language of the ‘battered child syndrome’ was broadly replaced by concerns around ‘non-accidental injury’, ‘child abuse’, ‘child protection’ and, eventually, ‘safeguarding’.

The battered child syndrome was a brief but important phenomenon of the 1960s, the study of which can offer three main conclusions. First, this article will challenge traditional narratives that the 1960s and 1970s saw the generalised and forced decline of the ‘golden age of medicine’, particularly by highlighting the role of radiologists themselves in inviting social agencies into the diagnosis of the syndrome.^6^ Second, the syndrome can inform the history of radiology and radiography. The historian Lisa Cartwright has emphasised the potential of studying the x-ray as a site of twentieth-century knowledge and power.^7^ The x-ray image was a powerful tool in raising awareness and concern around the battered child syndrome. However, numerous other medical professions also played important roles in co-constructing concerns around child maltreatment. An x-ray image must not be analysed in isolation, lest historians forget how a range of medical and social actors work together to produce, analyse and to justify the need for the radiological image. Third, this article will extend the analysis of the histories of secrecy and privacy in post-war Britain, outlined by Deborah Cohen in *Family Secrets* (2013). Cohen argues that the decades between the 1930s and the 1990s saw the vilification of secrecy.^8^ Whilst the x-ray could not visualise children's subjective experiences of pain, the application of this technology was key to providing an early challenge to the cultural denial and ignorance of child abuse. The battered child syndrome itself disappeared after the 1970s, but its legacy was the heightened concern around child abuse in Britain and America.

## Historiography

The significance of Kempe's article in heralding a ‘major breakthrough’ has been established by the social work scholars Nigel Parton and Harry Ferguson and by the historian of childhood Harry Hendrick.^9^ Concerns around child maltreatment had been present before the 1960s, and were primarily aired in the Victorian language of ‘cruelty to children’. However, cruelty to children did not evoke the same levels of public concern as child abuse does today, and was seen as primarily the responsibility of voluntary societies and philanthropists.^10^ The historian George Behlmer has written that as recently as 1880 English parents could ‘mistreat their children with virtual impunity’.^11^ The family home was thought of as a private and separate sphere where parents could discipline their children as they saw fit.^12^ Parton, Ferguson and Hendrick have demonstrated that Kempe's work encouraged medical practitioners to revisit their practice, and also drew the attention of journalists, politicians, and the public to broader issues of child maltreatment.^13^ In 1967 *The Times* newspaper went as far as to claim that before the invention of the battered child syndrome, the ‘medical profession, police, lawyers, and public’ had engaged in ‘self-deception’, desperately ignoring the violence perpetrated against children.^14^

Fuelling public and academic interest in Henry Kempe, the paediatrician's dramatic life story has recently been documented by his daughter Annie Kempe in the book *A Good Knight for Children: C. Henry Kempe's Quest to Protect the Abused Child* (2007). Kempe was born into a Jewish family in Germany on 6 April 1922. Following the rise of the Nazi party and the increasingly hostile environment towards the Jewish community Kempe travelled to America in February 1939. Having arrived as a ‘frail seventeen-year-old German-speaking immigrant’, Kempe nevertheless gained a Bachelor of Arts degree from the University of California in 1942.^15^ Kempe next attended the University of California Medical School where he developed an interest in virology. Kempe transferred his specialty to paediatrics in 1948, and was accepted as an Assistant in Paediatrics at the Yale University School of Medicine.^16^ At a welcoming party for new medical residents, Kempe met Ruth Svibergson, a fellow resident interested in paediatrics and child psychiatry. Kempe and Svibergson married just three months later.^17^ The Kempes would raise five daughters together, co-author several books on childhood, and found the National Centre for the Prevention and Treatment of Child Abuse and Neglect in 1972.

Whilst interesting, this article will not focus on the life, work or experiences of Henry Kempe. Rather, the article will assess the relationship between the invention of the battered child syndrome, the medical community and particularly the sub-discipline of radiology. The history of radiology has been increasingly well documented in recent years, notably by Adrian Thomas and Arpan Banerjee in *The History of Radiology* (2013), and within the journals of The British Society for the History of Radiology and the International Society for the History of Radiology.^18^ However, historians of radiology have not yet discussed the emergence of the battered child syndrome. The history of child abuse has also attracted increasing attention in recent decades, in response to contemporary alarms and awareness. Much academic attention has been paid by media theorists to various ‘moral panics’ around child abuse, for example the 1980s satanic ritual abuse scandals, and the Cleveland crisis of 1987.^19^ Further research has approached child abuse from a social policy perspective, including the work of Parton, Ferguson and Hendrick, or as a concern for contemporary social workers.^20^ However, these authors have not yet fully interrogated the role and significance of medicine, and particularly x-rays, in facilitating the early awareness of child abuse.

## The History of the Battered Child Syndrome

The first sustained medical investigation into child maltreatment was conducted by the French forensic pathologist Ambroise Tardieu in the mid-1800s.^21^ In 1860 Tardieu published his ‘Forensic study on cruelty and the ill treatment of children’, in which he highlighted thirty-two cases of physical abuse and neglect. Tardieu urged his medical colleagues that: ‘it remains that these cases are multiplying, that they provoke indignation, that they must not catch off guard the physician, often the only one capable of denouncing the crime to the legal authorities’.^22^ Tardieu later published further works on sexual abuse, physical abuse, neglect and infanticide.^23^ Even though France was a world leader in medicine at this time, however, Tardieu's work on child maltreatment had very little national or international impact. Indeed, Tardieu himself wrote in 1879 that his study of 1860 ‘did not draw attention’.^24^ People in the field of medicine, as well as in society more broadly, had not yet acknowledged the reality and prevalence of child abuse.

Indeed, as late as the 1960s ‘patently absurd’ explanations for child maltreatment were being offered at the University of Colorado Hospital, as observed by Kempe, and also by practising clinicians across the Western world.^25^ Doctors' refusal to accept that parents could or would wilfully harm their own children led them, consciously or unconsciously, to ignore the inconsistencies within children's case histories. The *British Medical Journal* (*BMJ*) testified in 1969 that the syndrome was ‘overlooked for many years’ because ‘the thought that one or other of the parents … could be responsible for its state is so repugnant to natural feeling that it does not come readily to mind’.^26^ Dr Henry Silver, a prominent American paediatrician, similarly later attested that ‘doctors were taught to believe what parents told them, and were usually convinced that parents were loving, and could never harm their children’.^27^ Without a substantial body of research around how to diagnose and to recognise beaten children, as exists today, parental explanations of childhood injury were accepted, and medical practitioners were even ‘taught’ to trust parents.

Indeed, radiology, which was to provide the first research on the diagnosis of beaten children, was in its infancy not only in America and Britain but globally in the early twentieth century. The initial discovery of x-rays is credited to the publication of ‘On a New Kind of Rays’ in 1895 by the Professor of Physics and Director of the Physical Institute at Würzburg University, Wilhelm Röentgen.^28^ In Britain and America, the following years were marked by great regional variation in the uptake of this new technology.^29^ The x-ray departments which did exist were often combined with electro-therapeutic departments and confined to poorly ventilated and damp cellars and basements.^30^ The historian Annie Jamieson has examined the slow adoption by British clinicians of x-rays and highlighted a variety of factors: that doctors felt able to adequately diagnose through physical examination, transillumination, cystoscopy and endoscopy; friction and protectionism within the medical profession; and lack of resources and poor organisation within institutions.^31^ The dissemination of x-ray technology was further impeded by the increasing publicity given to the hair loss, dermatitis and injuries regularly suffered by those operating these machines.^32^ Consequently, ‘the routine clinical use of X-rays lagged well behind the innovations reported in the medical and scientific press’.^33^

In his study of medical technologies, the historian Harry Marks highlights the x-ray as a technology particularly associated with the birth of a new medical specialty.^34^ However, there was a time lag between the invention of the x-ray and the creation of the specialties of radiology and radiography. In the British context, x-rays were used from the turn of the century but it was not until the 1920s that training courses were offered for the handling of x-ray equipment, and specialised ‘x-ray operators’ were unified by the Society of Radiographers.^35^ In 1930s Britain, doctors, rather than lay people, were increasingly appointed to operate x-rays, and the division between therapeutic and diagnostic operators of x-ray equipment was created.^36^

The practise of radiology and the professions of radiology and radiography expanded throughout the late 1920s, 1930s and 1940s in Britain and America. As radiology became increasingly popularised, the specific sub-discipline of paediatric radiology also began to emerge. The first ‘pioneer’ in paediatric radiology was the American Dr John Caffey. Caffey was born in 1895, the same year as the discovery of the x-ray; by the 1920s he was an established paediatrician, working as a private practitioner with ongoing ties to the Babies Hospital in New York City. Caffey authored or co-authored six articles on topics including platelet counts in children and meningococcal meningitis. In the early 1920s there was just one full-time paediatric radiologist in North America.^37^ At the Babies Hospital paediatric radiology ‘amounted to a twice-weekly visit from a radiographer’, and occasional radiology conferences. Following one such conference, convened by a pathologist rather than a radiologist, Caffey ‘remarked that it had been another hour wasted’.^38^ Caffey was overheard by the Babies Hospitals' chief of paediatrics who duly asked if Caffey himself believed that he could run the Department better. Caffey answered that ‘he could hardly do worse’.^39^ Subsequently, Caffey took charge of the Babies Hospital's radiology department. Paediatric radiology became Caffey's life's work, and he founded and edited the medical reference text *Pediatric X-Ray Diagnosis*, a crucial text in establishing and defining the intellectual basis of this field.^40^

In 1946, Caffey published an article in the *American Journal of Roentgenology* describing the cases of six infants admitted to the Babies Hospital with subdural haematoma, the pooling of blood under the skull. X-ray images taken of these children had revealed multiple long bone fractures in the children's arms and legs. There was no evidence to suggest that any of these children had a general or localised skeletal disease predisposing them to pathological fractures, and the children had no reported history of trauma. Nonetheless, Caffey insisted that ‘the fractures appear to be of traumatic origin’.^41^ With this article, Caffey invited radiologists and paediatricians to be more aware of the possibility that violence was, albeit occasionally, purposefully inflicted upon children. Caffey established himself, and the discipline of paediatric radiology, as radical, innovative and unafraid to draw what were, for this period, bold conclusions about child maltreatment. Authority in diagnosing the physical mistreatment of children by adults was thus concentrated within the professions of paediatrics and radiology. Building on Caffey's early work, these professions consolidated their status as ‘experts’ in managing child battering in both Britain and America over the following 20 years.

The next paediatric radiologist to consider beaten children was Dr Fred Silverman.^42^ Silverman received his paediatric training at Yale University before being trained at the Babies Hospital by John Caffey between 1945 and 1947, the years immediately following the publication of Caffey's aforementioned article. Silverman became the Director of the Division of Roentgenology at Cincinnati Children's Hospital in 1947.^43^ Here, Silverman published an article in the *American Journal of Roentgenology* in 1953 on the manifestations of skeletal trauma in infants.^44^ Silverman reported the cases of three infants with similar symptoms to those observed by Caffey. Silverman concluded that the bone fractures were clearly the result of several traumatic injuries. Consequently, he urged physicians to gain accurate patient histories, emphasising that parents may ‘permit trauma and be unaware of it, may recognize trauma but forget or be reluctant to admit it, or may deliberately injure the child and deny it’.^45^ The unwillingness of these studies to directly blame children's parents for causing these injuries was a result of the reluctance of radiologists to enter this fraught arena, and the training urging doctors to accept parental explanations. Caffey and Silverman's works were published seven years apart, a significant time to elapse in the medical press, and the interest of the medical community, at large, in child maltreatment was by no means fully secured in the mid-1950s.

Nonetheless, a body of radiological literature around child maltreatment developed between the mid-1950s and the early 1960s. Silverman's article was closely followed by the publication of the ‘Significance of Skeletal Lesions in Infants Resembling those of Traumatic Origin’ in the *American Medical Journal* in 1955, an article co-authored by Dr Paul Woolley from the Department of Paediatrics at the Children's Hospital of Michigan and Dr William Evans of the College of Medicine at Wayne State University.^46^ Woolley and Evans offered the first retrospective study in this area, analysing the records of children admitted to the Children's Hospital of Michigan between 1946 and 1954. Their study found ‘no consistent evidence to suggest wide variation in bone fragility among infants’.^47^ Furthermore, they emphasised that when these infants were removed from their environments for periods varying from a week to several months no new lesions developed.^48^ Woolley and Evans drew attention to what they termed ‘aggressive, immature or emotionally ill’ parents, and claimed that the circumstances and motivations of the parents must be taken into account when considering the injuries of children.^49^ Woolley and Evans' article did not explicitly state that some parents were violent towards their children, again emphasising the radical and difficult nature of making such a statement at this time. Nonetheless, the authors' conclusions that child bone fragility was consistent, that new injuries did not develop outside of the parental home and that the social and psychiatric motivations of parents must be examined in making diagnoses, all gestured towards the idea of familial violence. Indeed, in 1968 the paediatricians Robert Bensel and Samuel Radbill, and the mass communication theorist Marguerite Rheinberger, went so far as to retrospectively suggest that Woolley and Evans' article ‘blasted the medical profession … for its reluctance to concede that the multiple injuries to children were committed wilfully’.^50^

Child maltreatment was being considered by a small but significant number of paediatricians and radiologists, all in America, in the first half of the twentieth century. Building upon this small but important and growing body of literature, the paediatrician Henry Kempe was the first doctor to explicitly detail and examine child battering at length.^51^ At the age of thirty-four Kempe was appointed as the Chairman of the Paediatrics Department at the University of Colorado. In this role, his interest in undiagnosed child abuse peaked. In a letter to a friend Kempe stated that ‘I got into the problem of the battered child for no reason of altruism but rather, at first, out of rage at the intellectual blocks I encountered when I went on ward service in 1957 and 1958.’^52^ Like other medical professionals, Kempe struggled to comprehend the idea that parents could purposefully harm their own children. Nonetheless, he could not ignore the clear dissonance often apparent between parental explanations and medical evidence and became highly critical of doctors who insisted on offering implausible explanations for non-accidental injuries.^53^

Kempe published ‘The Battered Child Syndrome’ in the *American Medical Association Journal* in 1962. The article drew authority from Kempe's seniority at the University of Colorado, and from the eminence of his co-authors; the University of Colorado Professor of Paediatrics Henry Silver, the Associate Professor of Psychiatry Dr Brandt Steele, and the Assistant Resident in Obstetrics and Gynaecology Dr William Droegemueller. The final co-author was Fred Silverman, of the Children's Hospital, whose contribution to the developing awareness of violence against children during the 1950s has already been highlighted. The collaboration between Silverman and Kempe again emphasises the small number of physicians who were publishing on this controversial and difficult topic in this era. Silverman, it seems, had recognised that parents could behave violently towards their children when publishing in 1953. However, he had not yet felt ready or prepared to explicitly document this phenomena. Whilst Kempe's co-authors are less often remembered than Kempe, the support of such eminent scholars was crucial to the dissemination and acceptance of this radical article. The scholars came from a variety of medical professions which emphasised, from the outset, that the complex nature of the battered child syndrome would require the attention of all physicians. The co-authorship of the psychiatrist Brandt Steele, particularly, directed debate towards the psychiatric and social characteristics of parents, an important aspect of the syndrome first identified by Woolley and Evans. This focus on multiprofessional action empowered numerous medical professions in Britain, including paediatrics and radiology but also dermatology and ophthalmology, to seek to explain how to define and manage the battered child syndrome.

Kempe, Silver, Steele, Droegemueller and Silverman defined the ‘battered child syndrome’ to:

characterize a clinical condition in young children who have received serious physical abuse, generally from a parent or foster parent. The condition has also been described as ‘unrecognized trauma’ by radiologists, orthopedists, pediatricians and social service workers, and is a significant cause of childhood disability and death. Unfortunately, it is frequently not recognized, or, if diagnosed, is inadequately handled by the physician because of hesitation to bring the case to the attention of the proper authorities.^54^

The authors hoped to understand the incidence of the battered child syndrome by conducting a year-long nation-wide survey. During this survey 71 hospitals reported 302 cases to the authors, and 77 District Attorneys reported 447 cases. Using this data, the authors drew preliminary conclusions around the psychiatric condition of the battering adult, offered two examples of ‘typical cases’ and explained potential techniques with which to evaluate the syndrome, focusing upon its radiologic features. The syndrome might be present, the authors explained, in any child exhibiting evidence of fractured bones, subdural haematoma, failure to thrive, soft tissue swellings or skin bruising, or a child who died suddenly, or whose degree or type of injury was at variance with the history given by the occurrence of the trauma exhibited. With this article, Kempe and his co-authors dramatically asserted that it was ‘the physician's duty and responsibility to the child’ to no longer ignore this syndrome, but to provide ‘a full evaluation of the problem and a guarantee that the expected repetition of trauma will not be permitted to occur.’^55^ The article states that other than ‘extremely sociopathic characters’, or if a non-abusive spouse reported an abusive spouse, ‘more often there is complete denial of any knowledge of injury to the child and the maintenance of an attitude of complete innocence of the part of both parents’.^56^ Rather than automatically believing the explanations offered by parents, the article urged, physicians held a ‘duty’ to conceptualise the child as their patient and to thoroughly, and without parental influence, consider the origin of the child's injuries. In later years, Kempe argued that the physicians ‘duty’ reflected a child's rights, drawing on broader rights discourses of the late 1960s and particularly the 1970s. He told *The Times* in 1970 that: ‘We think far too much of the “rights” of the parents and not enough of the “rights” of the child.’^57^

It was highly radical to question the role of parents in explaining and diagnosing a child's injuries in the 1960s. Indeed, it was not until the 1990s that paediatricians more broadly began to question whether children should be treated as ‘healthcare consumers’, with their preferences and voices considered above those of their parents.^58^ Thus, the authors of the battered child syndrome article called for a disregard of parental preferences some 30 years earlier than the majority of paediatricians, presumably because doctors were more ready to disregard the voices of violent, rather than non-violent, parents. The paediatricians of the 1990s, bolstered by the findings of child psychiatrists in the 1980s that children could be highly informed and rational from a young age, urged clinicians to consider the child's voice, and to ask children how they preferred to be treated. By contrast, Kempe and his co-authors did not advise directly asking children whether they had been mistreated. Kempe suggested that the battered child, who was usually under three years of age, would lack the rationality and the confidence to properly describe the origins of his or her injuries.^59^

Instead, Kempe and his co-authors believed that it was possible to glean an objective and clear measure of whether a child had been beaten by using x-rays. Their article stated that ‘when parental assault is under consideration, radiologic examination of the entire skeleton may provide objective confirmation’.^60^ Similarly, writing in a later collection on the battered child, Silverman emphasised that the radiologic conditions of the syndrome were ‘surprisingly specific’.^61^ Caffey told the American Paediatric Society in 1965 that the distinctive characteristics of growing bones meant that x-rays could provide ‘conclusive evidence’ of whether a fracture had been caused by trauma. Children who had been regularly beaten would display ‘traumatic involucrums’ at several stages of bone development when x-rayed.^62^ The initial ‘Battered Child’ article contended that many physicians were reluctant ‘to accept the radiologic signs as indications of repetitive trauma and possible abuse’.^63^ ‘This reluctance’, Kempe and his co-authors stated, ‘stems from the emotional unwillingness of the physician to consider abuse as the cause of the child's difficulty and also because of unfamiliarity with certain aspects of fracture healing’.^64^ Nonetheless, the article continued: ‘To the informed physician, the bones tell a story the child is too young or too frightened to tell’.^65^

Such an attempt to ‘read’ the human body with medical machinery has a long history. Stanley Joel Reiser has demonstrated that, since the early nineteenth century, physicians have sought to ‘inspect the architecture of the internal organs during life with the ease and clarity possible after death’.^66^ Visual diagnostic techniques began to compete with aural ones in this century, first as doctors crafted instruments to look within the human eye and, later, machinery to observe the larynx.^67^ The historian of medicine Joanna Bourke has similarly traced how physicians have throughout history been keen to find ways to measure and to objectify pain, whether through surveying tools such as the McGill Pain Questionnaire, Infrared Imaging Thermography, or the recent ‘holy grail’ of objective detection and measurement of pain, brain imaging.^68^ These inventions, Reiser writes, would allow practitioners to ascertain the veracity of their patients' complaints. For example, doctors could now tell if people were seeking to avoid military service by feigning nearsightedness.^69^ In the case of the battered child syndrome, the x-ray would not be used to uncover lies but, rather, to reveal truths which children themselves could not tell. Indeed, physicians have remained keen to find a medical tool which can signal, to an informed practitioner, if a child is being abused, and in the 1980s, paediatricians in Britain and America sought to use reflex anal dilatation as a diagnostic tool for child sexual abuse.^70^

By the 1970s, practitioners recognised that x-rays did not provide an objective measure of child abuse, but rather must be used merely as one indicator alongside evidence from family case work and interviews. Caffey cautioned the American Paediatric Society about the limitations of x-rays in 1965. He stated that: ‘It cannot be emphasized too strongly, however, that even classical radiographic changes of trauma in the bones, tell nothing of the person who abused the child or how it was abused’.^71^ X-rays could demonstrate if a child's injuries were caused by trauma, but they could not tell physicians who had inflicted trauma on the child. Caffey stated that suspicion must thus be cast not only on parents but also visiting aunts and uncles, grandparents, cousins, siblings, babysitters, nurses and housemaids.^72^ The x-ray could help doctors seeking to protect children, but were not a ‘magic bullet’. The field of child protection was to remain fraught, contentious and difficult.

In Kempe's article, knowledge of the syndrome was, on the one hand, directed specifically to ‘informed’ radiologists and paediatricians. The battered child syndrome was, through the ‘objective confirmation’ of the x-ray image, to be diagnosed and shaped by American radiologists. However, on the other hand, authority in managing the syndrome was also bestowed upon the medical community at large, designated by the term ‘syndrome’ and implied by the range of professionals who contributed to Kempe's article. Kempe did not work alone, but alongside psychiatrists, obstetricians, gynaecologists, radiologists and other paediatricians. Caffey more broadly indicated that advice must be sought from social sources, outside of medicine, to ascertain who had hurt a child. The philosopher of science Ian Hacking has suggested that the very word ‘Kempe’ is now perceived to represent not only a man but also ‘a radical break in our awareness’.^73^ Certainly, Kempe was significant in consolidating previous research findings around child maltreatment under the title of ‘battered child syndrome’. Kempe himself stated that he created this term as a ‘jazzy title, designed to get physicians’ attention'.^74^ In later years the *American Medical Association* recognised the ‘Battered Child Syndrome’ paper as one of the 60 most important published medical manuscripts of the twentieth century.^75^ The creation of this term directed medical, public, political and media interest towards child abuse; an area of concern which would continue to develop and grow even as the specific term ‘battered child syndrome’ fell out of use from the late 1960s. Nonetheless, historians must not overstate the significance of Kempe himself to the detriment of considering the network of professionals who contributed to, and also appropriated and redeployed, his original work, constructing and changing its social and political impact in the process. Indeed, once published, Kempe's article was quoted, utilised and mediated by a range of medical actors seeking to diagnose this syndrome with the tools of their profession; as I will demonstrate by examination of how the syndrome emerged, developed and disappeared in the British context.

## The Syndrome in Britain

The field of radiology has always progressed in an international context. The need to share discoveries, inventions and research across national boundaries was formally recognised by the foundation of the International Society of Radiographers and Radiological Technologists in 1962. By 2013 this organisation encompassed 71 national radiographic societies from 68 countries and had more than 200,000 members.^76^ Work in America has previously influenced that in Britain, for example Thomas and Banerjee highlight how the first female radiologist, Florence Stoney, brought a Coolidge X-ray tube from America to Britain in October 1914.^77^

Medical professionals, and particularly radiologists, played a very important role in transferring knowledge around the battered child syndrome from America to Britain. Indeed, the first discussion around the battered child syndrome in the British context was in 1963, just one year after ‘The Battered Child’ was published, and arose within the *British Medical Journal*. In this journal, D. Li Griffiths and F. J. Moynihan, orthopaedic surgeons at Manchester University and Guy's Hospital, London, respectively, published an article entitled ‘Multiple Epiphysial Injuries in Babies (“Battered Baby” Syndrome)’. Having become aware of Kempe's work, these surgeons aimed ‘to give publicity to a syndrome which we think commoner than is usually believed’.^78^ The article's characterisation of the syndrome fell broadly in line with Kempe's original description, and three further case studies were offered. Like Kempe, Griffiths and Moynihan acknowledged that ‘doctors are reluctant to believe that such assaults on innocent babies are possible’. Nonetheless, Griffiths and Moynihan wrote, ‘in the interests of some of our most helpless patients we must realize that epiphysial trauma is due to violence and that not all parents, even if warned, are safe custodians’.^79^ Thus, these surgeons, following the writings of the American radiologists on the battered child syndrome, highlighted the need to interrogate parental explanations for children's injuries.

The National Society for the Prevention of Cruelty to Children later claimed that Griffiths and Moynihan's article ‘aroused considerable interest and led to an increasing awareness that the problem was far more common than had previously been realised’.^80^ The article certainly generated much debate within the *British Medical Journal* itself, where many physicians submitted letters noting that the battered babies whom Griffiths and Moynihan had highlighted may only represent the ‘tip of the iceberg’, and advocating the urgent need for further research.^81^ Following Silverman's assertion that the syndrome was ‘surprisingly specific’, E. E. Sumpter, of the Paddington Green Children's Hospital, wrote to the *British Medical Journal* in 1966 to emphasise that ‘the Battered Baby (Social) Syndrome can be precisely defined as medical’.^82^ The syndrome was conceptualised to have ‘symptoms’, rather than ‘signs’, and to be ‘diagnosed’, not ‘identified’. Sumpter contrasted the syndrome to child abuse, child neglect and child ill-treatment, which, she believed, were social issues which could not be diagnosed nor treated by medical practitioners.^83^ By the mid-1960s, there was broad consensus amongst the British medical community that the battered child syndrome was a medical condition worthy of further inquiry. In 1965 the syndrome was included within both the definitive British textbook of paediatrics, *Disease in Infancy and Childhood*, and the index of medical journals, *Index Medicus*.^84^ In 1966 the British Paediatric Association published a memorandum on the recognition and management of the condition.^85^

Research around the battered child syndrome conducted in America was rapidly adopted in Britain not only by medical professionals but also by the NSPCC. Following a meeting with Kempe in Colorado in 1964, the Director of the Society, Reverend Arthur Morton, became ‘very keen that the society should be in the forefront of the work needed to counter’ the syndrome.^86^ Under Morton's guidance the Battered Child Research Unit was established in 1966.^87^ The Unit was established in ‘Denver House’, an explicit tribute to Kempe's work in Denver, Colorado. The primary aim of the Unit was to ‘create an informed body of opinion about the syndrome and to devise methods of treatment’, and the work produced there gained Parliamentary and media attention.^88^ Ian Hacking and the historian Phillip Jenkins have suggested that the NSPCC acted as a ‘conduit’, ‘exporting’ the battered child syndrome into Britain.^89^

Public policy transfer literature has characterised a ‘simple bilaterial exchange’ of policy and practice from one sovereign state to another as ‘methodological nationalism’.^90^ However, whilst the transfer of knowledge around the battered child syndrome was initially unidirectional, British paediatricians, radiologists and the NSPCC began to conduct their own landmark research by the mid-1960s. At this stage, British and American experts began to share and exchange their research findings in discussions that concurrently altered the working conceptions of the battered child syndrome in both countries. Public policy literature has termed such interactions, where professionals in different countries simultaneously act to co-construct change, ‘transnational transfer networks’.^91^ A number of individuals were particularly important in facilitating the exchange of knowledge around the battered child syndrome between Britain and America. Arthur Morton and Henry Kempe each spent time working within both countries.^92^ Similarly, Professor Brandt Steele, and Doctors Carl Pollock and Ray Helfer, American contributors to the *The Battered Child*, an edited collection of research studies, each held many discussions with the NSPCC.^93^ Public policy theorists Martin De Jong and Jurian Edelenbos have termed individuals who personally diffuse information between national contexts through direct social contact ‘transfer agents’.^94^ These ‘transfer agents’ did not adapt their characterisations of the syndrome to the different national contexts of Britain and America, despite clear differences between the health care systems of both countries. Indeed, Kempe defended the idea that his research could be straightforwardly adopted in both countries, and told *The Times* in 1970 that: ‘There seems to be no differences whatsoever between the way parents and children interact in our two countries. The best is very good and the worst is terrible.’^95^

The most significant outcome of these international discussions was not in changing the specific construction of the battered child syndrome, but in enabling a small and disparate group of transfer agents to mutually reassure one another of the fundamental necessity for further research into child battering. This follows the conclusions of the international relations scholar Diane Stone, who argues that transnational co-operation can create new norms as well as changing ideas, policy instruments and practices.^96^ During the early 1960s the need to research the battered child syndrome in the British context was primarily advocated by radiologists, orthopaedic surgeons, paediatricians and the NSPCC. The topics of the battered child syndrome and child abuse had not yet gained particular attention from politicians, media nor public. Hence, by sharing knowledge about the extent and characteristics of the syndrome, these transnational transfer agents became increasingly convinced of the importance of disseminating their own research in both Britain and America.

In Britain, medical writings around the syndrome went so far as to suggest that doctors held a ‘moral and legal’ ‘duty’ to recognise the battered child syndrome.^97^ The language of a ‘moral duty’ may have been shaped by the context of the post-war government's acceptance of a duty to care for all of its citizens under a national health service. ‘Moral duty’ suggested that children were due, or owed, the attention of their doctors to cases of child maltreatment, and continued research into the battered child syndrome. The idea that clinicians owed significant and unavoidable duties to children reflected the broader ideas that the emergent welfare state, social policy and also the education system must protect the valuable and important life stage of childhood in the post-war era.^98^ Aside from the language of duties, the early medical writings on child battering were also laden with emotive and moralistic language.^99^ The *British Medical Journal* declared that ‘these cases are among the most unpleasant’ and ‘evil’ that ‘a doctor has to deal with’ and that ‘there is a distinctive nightmare quality in this total situation’.^100^ The publication of such evocative language was very unusual within this journal, and perhaps sought to emphasise doctors' shock at having just realised that some parents beat their children, and their remorse for having overlooked child maltreatment until this time. Thus, towards the mid- to late 1960s the British medical profession began to vocally and emotively present itself as being at the forefront of research into the battered child syndrome.

Amongst the medical profession at large, disagreement soon arose around the specific symptoms to consider, and the sub-profession best suited to identifying those symptoms. The syndrome was initially defined in very vague terms, with practitioners only able to universally agree that the syndrome reflected systematic parental violence against very young children. The British Paediatric Association's memorandum, for example, provided the general explanation that ‘the ‘battered baby syndrome’ is a name given to a collection of symptoms and signs occurring in children who have suffered repeated injuries at the hands of their parents or others … most of these children are probably under two years of age’.^101^ The diagnosis of the syndrome was restricted to very young children because older children were thought to be capable of giving a reliable history of their own injuries.^102^ Some physical features of the syndrome were repeatedly recognised in British medical works, particularly those initially identified by Kempe: fractured bones, subdural haematoma, failure to thrive, soft tissue swellings, skin bruising and a child who dies suddenly.^103^ However, these characteristics were merely a list of the features regularly associated with the syndrome, rather than a systematic and definitive list of identifiers. As such, a ‘problem of definition’ remained with clinicians interested in, but struggling to coherently characterise, the battered child syndrome.^104^ In this context, a diverse range of medical professions sought to capitalise upon the desire for objective, definitive and authoritative ‘symptoms’ by positioning their own professions as uniquely best placed to characterise the battered child syndrome.

In a *British Medical Journal* article of 1967, for example, Michael Gilkes, of the Sussex Eye Hospital, emphasised the ocular conditions connected with the syndrome, claiming that in cases where the battered child was being diagnosed through the appearance of a subdural haematoma the presence of gross fundus appearances, to be diagnosed by ophthalmologists, would ‘considerably increase one's suspicions’.^105^ Suzanne Alexander, from the Institute of Dermatology in London and surgeon Andras Barabas, similarly both emphasised their own professions’ roles in differentiating between purposefully inflicted bruises and infants particularly prone to bruising due to the existence of an underlying condition (such as the Ehlers-Danlos syndrome).^106^ Despite the publications of ophthalmologists and dermatologists, however, radiologists gained the most success in emphasising the ability of the x-ray to detect the battered child syndrome.

Thomas and Banerjee identify the 1960s as a period in which radiology ‘was becoming increasingly complex and expensive’ due to the ongoing invention of new technologies for x-ray departments.^107^ In this context, British radiologists entered debates around the battered child syndrome to lobby for the increased provision of resources to their poorly funded departments.^108^ Numerous radiologists wrote to the *British Medical Journal* underlining that limited funding hindered the provision of x-rays to potentially battered children, which enabled the re-battering of children wrongly returned to their parents.^109^ These radiologists positioned their research into the symptoms and management of the syndrome as potentially able to save children's lives, and dramatically improve children's well-being.

**Fig. 1:**
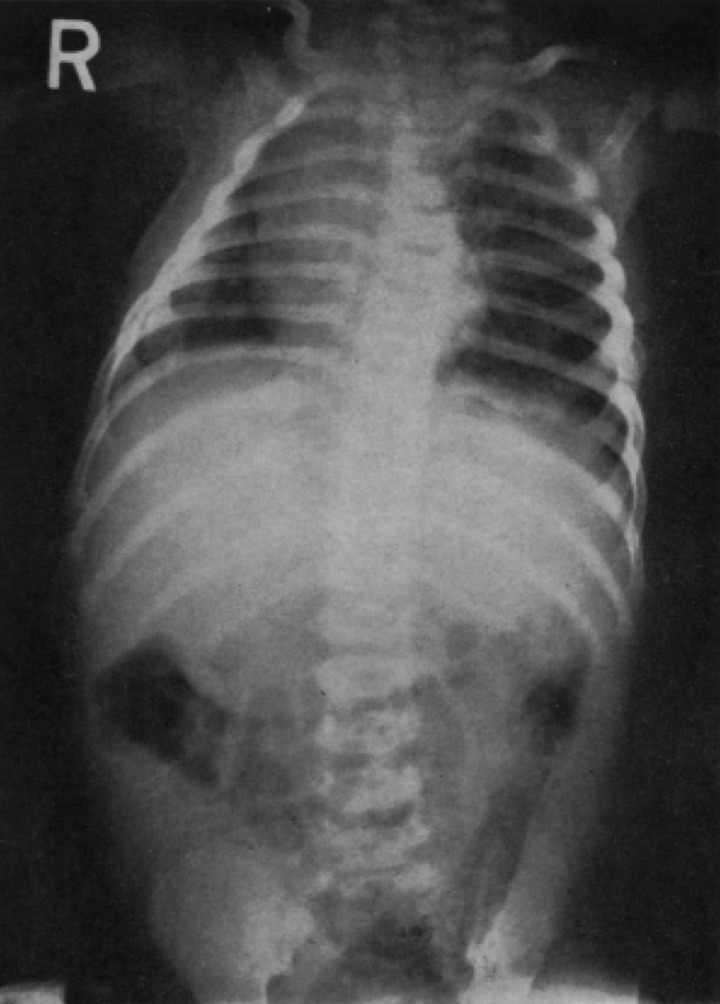
X-ray showing healing fractures in seven ribs of a two-month-old baby.

*Source*: D. Li Griffiths and F. J. Moynihan, ‘Multiple Epiphysial Injuries in Babies (“Battered Baby” Syndrome)’, British Medical Journal, 21 December 1963, 1560.

**Fig. 2:**
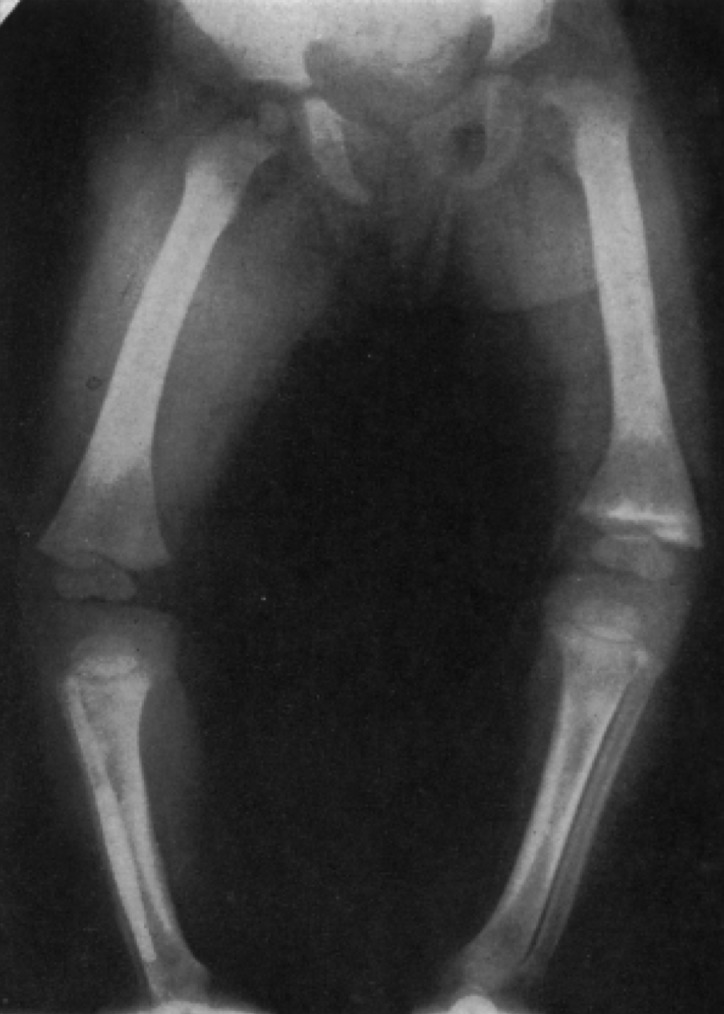
X-ray showing partial separation of a left upper femoral epiphysis.

*Source*: Griffiths and Moynihan, ‘Multiple Epiphysial Injuries in Babies’, 1560.

Keen to prove that the battered child syndrome held distinct and unusual radiological features, radiologists provided medical journals with numerous x-ray images. Two of the images provided by Griffiths and Moynihan in their original article are provided in Figures 1 and 2.

By offering these images, Griffiths and Moynihan challenged their peers who continued to deny the reality of violence against children and, simultaneously, emphasised that x-rays could provide a way to make the unreported injuries of children, and the invisible violence of parents, detectable. Figure 1 portrays healing fractures in seven ribs of a baby of just two months old. Figure 2 represents the partial separation of a left upper femoral epiphysis. These fractures were not reported by the children's guardians, and without x-ray technology such injuries could not be identified by medical professionals or articulated by the young children themselves. Indeed, Figure 2 shows new bone beginning to form around the upper end of the child's leg shaft; this displays the added difficulties of diagnosing invisible injuries when children's adaptable bodies enable them to heal themselves from many physical traumas without medical intervention. These images received much debate within the *British Medical Journal*, and indeed the evocativeness of images as a persuasive tool with which to disseminate information about health has been attested to within the growing literature around visual culture and the history of medicine.^110^ The notion that these physical images could provide a failsafe and unquestionable measure of the battered child syndrome was an alluring one to the medical profession. The practice of radiology was standardised in the 1950s, as international guidelines for deploying this technology were agreed upon.^111^ In addition to providing an objective measure, x-ray images, unlike verbal or textual descriptions, could also be easily utilised within all hospitals and medical establishments throughout the United Kingdom, providing a uniform tool with which to diagnose the battered child. The potential of the x-ray received some media attention also. For example, in 1966, *The Times* emphasised that battered babies may have as many as 60 or 70 injuries; some of which could only be revealed by x-ray.^112^

Discussions around the battered child syndrome towards the late 1960s saw the monopoly of the medical profession in defining the syndrome break down. This challenge to medical authority was led by medical practitioners themselves, who increasingly recognised that the distinction between the social issues of child abuse and the medical issues of the battered child syndrome could not be maintained in practice.^113^ Physicians recognised that social problems were causing and exacerbating, and were hence key to understanding, the roots and existence of the syndrome. Additionally, many doctors acknowledged that they must work alongside social workers, police and health visitors to detect and help battering families. Signifying this, in partnership with the paediatric educator Ray E. Helfer, Kempe created the edited collection *The Battered Child* in 1968. The collection contained chapters written by a social worker, a welfare department worker, a lawyer and a policeman, as well as contributions from a radiologist, a paediatrician and a psychiatrist. As such, the collection thus highlighted the unique contributions which could be made by ‘many disciplines involved in helping the battered child and his parents’.^114^ One chapter emphasised that the prevention of battering required the creation of ‘an atmosphere in the community … which is not conducive to overlooking violence’.^115^ Whilst radiology continued to play a role in the management of the syndrome, x-rays were no longer thought to provide a clear, singular, and objective diagnosis of child abuse. In parliamentary attention subsequently paid to child abuse in Britain, the x-ray was to be one of many diagnostic aids, alongside other medical measures such as retinal examination and analysis of bruising but also thorough discussion with parents and children.^116^

## Conclusion

The social history of medicine has spawned many writings surrounding the changing nature of medical power and authority from antiquity to the modern day.^117^ Historians of medicine have written about the ‘golden age of medicine’, a time in which medical professionals enjoyed high levels of social prestige and public trust.^118^ There is, however, substantial disagreement amongst historians of medicine around the ‘distinct moment of inception’ of this ‘golden age’; Allan M. Brandt and Martha Gardner, for example, suggest that the golden age consisted of the late nineteenth century and early twentieth century, whilst Mike Saks highlights the first half of the twentieth century, and Edward Shorter and Susan Lawrence emphasise the middle third of the twentieth century.^119^ This latter ‘golden age’ was fuelled by the discovery of numerous new ‘wonder drugs’, such as penicillin, cortisone and Prontosil.^120^ Concurrently, contemporary journalists bestowed positive coverage upon successive developments in medical technologies including kidney transplants, hip replacements and test tube babies. In this context, Shorter has emphasised that medical professionals were seen as ‘demigods in white’.^121^ The training of these ‘demigods’, Lawrence has argued, drew legitimacy from its heavy focus on the scientific basis of disease and cure.^122^

During the 1950s, 1960s and 1970s continuing specialisation amongst medical professionals and the ongoing development of medical technologies and pharmaceuticals made the practice of medicine seem more competent than ever. Historians have identified as ‘one of the great ironies of the social history of medicine’ that, despite these developments, new and unprecedented challenges questioned medical authority in this era.^123^ Diminishing levels of public trust in the medical establishment were expressed through the rising number of malpractice suits, declining loyalty towards specific medical providers, and the flight to alternative therapies.^124^ Challenges to medical authority were compounded by the slowing development of drugs and technological innovations, the rising costs of medical care amidst the economic downturn of the 1970s and the decline of clinical science.^125^ Historian Catherine Crawford has explored the role of the law and legal profession in raising questions over the ‘demigod’ status of the medical profession in this era. The 1960s and 1970s marked, she claims, greater legal scrutiny of medical negligence, new questions around informed consent, and mounting evidence of unequal access to the resources of health care delivery systems.^126^ Challenges to medical power were further compounded by broader social questions around the authority of ‘experts’ in this era, as the Vietnam War spawned a ‘protest generation’ and trade unions began to attack the professions.^127^

Declining public trust during these years expedited a declining confidence within the medical profession itself. This provides the context for the uncertain and confused attempts by British radiologists, orthopaedic surgeons, ophthalmologists, dermatologists and paediatricians to definitively conceptualise the battered child syndrome. Despite disagreements amongst the medical community, however, the ‘battered child syndrome’ was ‘invented’ by medical practitioners and researchers. A range of individuals played a crucial role in bringing the syndrome to the attention of the public and social work professionals. Hence, claims that medical authority declined in the 1960s must be tempered by the recognition that this decline was by no means rapid nor instantaneous. Furthermore, the role of medical professionals themselves in contributing to a deconstruction of the ‘golden age’ of medicine must also be recognised, at least in the case of the battered child syndrome. Kempe initially ‘discovered’ this condition and positioned radiologists as the primary diagnostic agent. However, by the late 1960s he had reconceptualised x-rays as merely one diagnostic tool amongst many, and also recognised the need to work with social agencies in order to alleviate child abuse. Medical authority was both authoritative and uncertain, relied upon and questioned, trusted and suspected by the public, social welfare professionals and factions of the medical profession themselves during the 1960s. These contradictions became particularly apparent during the widespread debates following the ‘invention’ of the battered child syndrome.

The relationship between radiology and the syndrome has not yet been assessed by historians of child abuse nor of radiology, despite the fact that radiologists were particularly successful in negotiating themselves a key role in the identification of child battering. Kempe's article drew upon a small but significant body of radiological literature which was produced as the profession of paediatric radiology was new and growing. In Britain, the publication of ‘The Battered Child Syndrome’ saw radiologists seek to direct funding and resources to their increasingly expensive profession. Nonetheless, the influence and significance of the battered child syndrome cannot be fully understood through consideration of the x-ray and radiology alone. In *Screening the Body* (1995), Cartwright claims that:

the X ray is a major technique of twentieth-century medical knowledge and power. It has been a war machine, used in national battles against disease and in battles for global prominence in science; and it also has been a metaphorical site of major importance. The X-rayed body, stripped of its overinscribed gender- and race-encoded epidermis and organs, is an apt figure both for the nightmare of eugenics, with its agenda of eradicating some body types, and for the utopian fantasies of a social order no longer predicated on typologies on the organic body.^128^

Radiologists hoped that x-rays would provide a clear visual indication of whether a child had been abused. The x-ray presented and could benefit any and all children, regardless of race, gender or class. However, at the same time, considering the battered child syndrome from the perspective of radiology alone will not fully capture how the syndrome was co-constructed by a range of medical actors. The authority of the x-ray to provide the primary form of diagnosis was challenged, for example, by dermatologists and ophthalmologists, who sought to assess the syndrome through the skin and the eyes, respectively. Even the x-ray itself was ordered, analysed and assessed not only by radiologists but also by paediatricians, general practitioners and accident and emergency staff. The x-ray provides a significant and fascinating image, but its significance is negotiated by a complex network of medical actors.

The historian Deborah Cohen emphasises that the post-war era saw the ‘entangled histories of secrecy and privacy’ separate.^129^ Before the twentieth century, Cohen asserts, secrecy forged trust between family members and was seen as the ‘indispensible handmaiden’ of privacy for the various sorts of shame that could be visited upon families—an illegitimate birth, a son with a propensity for ‘unnatural crimes’, suicide, insanity, adultery, bankruptcy'.^130^ However, ‘as privacy was written into law in Britain between the 1930s and the 1990s, secrecy was ever more vilified’.^131^ Secrecy, as a ‘familial strategy for reckoning with disgrace or misfortune’ began to be viewed as ‘destructive, a malign practice that erodes trust, especially between family members’.^132^ Child abuse, previously ignored, denied, and left within the privacy of family homes, was seen as a dangerous and destructive evil that the public needed to take responsibility for acknowledging and preventing. The lines between acceptable discipline within the privacy of the familial home and unacceptable child battering were drawn. Lifting the secrecy around child abuse, after all this time, would not be easy and, in this context, the technology of x-rays seemed to provide an objective and authoritative measure through which to determine whether a child had been subjected to violence. Ultimately, x-ray images could not represent the social mistreatment which many children were subjected to. X-ray images require an interpretation, the subjective construction of a ‘story’ between radiologist and image, not the objective telling of ‘a story the child is too young or too frightened to tell’.^133^ Nonetheless, in its brief lifespan the battered child syndrome functioned to challenge the secrecy around child maltreatment, and the syndrome left in its wake increased medical, social and political concerns about child abuse and child protection.
